# Supplementary material for: MetaBinner: a high-performance and stand-alone ensemble binning method to recover individual genomes from complex microbial communities
Source: Genome Biol. 2023 Jan 6;24:1. doi: 10.1186/s13059-022-02832-6 (PMC9817263; doi:10.1186/s13059-022-02832-6)
Supplement: Supplementary file 1 — Additional file 1. Supplementary Materials. It includes descriptions about the ground truth annotations provided by CAMI II challenges (Section A), a brief description of k-means++ method (Section B), descriptions about the parameters used in Step 4 of MetaBinner (Section C), Fig. S1 and Tables S1-S4, and commands for executing the compared binning methods and assemblers (Section F). [file 13059_2022_2832_MOESM1_ESM.pdf]

Supplementary Materials for "MetaBinner: a high-performance and stand-alone ensemble binning method to recover individual genomes from complex microbial communities"

Ziye Wang[1,2], Pingqin Huang[3], Ronghui You[1], Fengzhu Sun[4], and Shanfeng Zhu[1,5,6,7,8]✉

[1] The Institute of Science and Technology for Brain-inspired Intelligence, Fudan University, Shanghai, China
[2] School of Mathematical Sciences, Fudan University, Shanghai, China
[3] Shanghai Key Lab of Intelligent Information Processing, School of Computer Science, Fudan University, Shanghai, China
[4] Department of Quantitative and Computational Biology, University of Southern California, Los Angeles, CA 90089, USA
[5] Shanghai Qi Zhi Institute, Shanghai, China
[6] Key Laboratory of Computational Neuroscience and Brain-Inspired Intelligence (Fudan University), Ministry of Education, Shanghai, China
[7] MOE Frontiers Center for Brain Science, Fudan University, Shanghai, China
[8] Zhangjiang Fudan International Innovation Center, Shanghai, China
`zhusf@fudan.edu.cn`

A Descriptions about the ground truth annotations provided by CAMI II challenges

The ground truth annotations of contigs from the simulated datasets for AMBER are provided by the organizer of CAMI I and II challenges (https://data.cami-challenge.org). The simulated datasets were generated with CAMISIM (https://github.com/CAMI-challenge/CAMISIM). As introduced in [3,18], CAMISIM generates a BAM file for each sample of a dataset, which gives the alignment of simulated reads to reference genomes. Furthermore, it extracts the perfect co-assembly of all samples by including all regions covered by at least one read according to the BAM files. The perfect co-assembly is used for binning, named as "gold-standard cross-sample assembly" in our paper, as done in [4]. Strain-level is used for measuring the performances of tools using AMBER.

B Brief description of k-means++

K-means++ [42] is a variant of k-means, which utilizes a smart way to initialize clustering centers to improve clustering accuracy and computational speed. K-means++ uniformly chooses a data point as the first initial center c_1 at random from the set of data points, $\mathcal{X}$. A new center c_i is chosen from $\mathcal{X}$ with probability $P(x)$, which is defined in equation (1). The process is repeated until K centers have been chosen.

$$P(x) = \frac{D(x)^2}{\sum_{x\in\mathcal{X}} D(x)^2}, \tag{1}$$

where $D(x)$ is the shortest distance between point x and the closest previously chosen initial center.

C Parameters used in Step 4 of MetaBinner

Step 4 is developed for generating more low-contamination bins for further integration by splitting bin with high contamination. According to the definitions for estimated completeness and contamination given in CheckM, it is possible for a bin with high contamination (>50%) and low completeness (<50%). Therefore, we also set a high completeness condition to split bins to avoid unnecessary waste of time on the bins with low completeness. We further tried different values of parameters for MetaBinner in CAMI Airways, as shown in Additional file 1: Table S4.

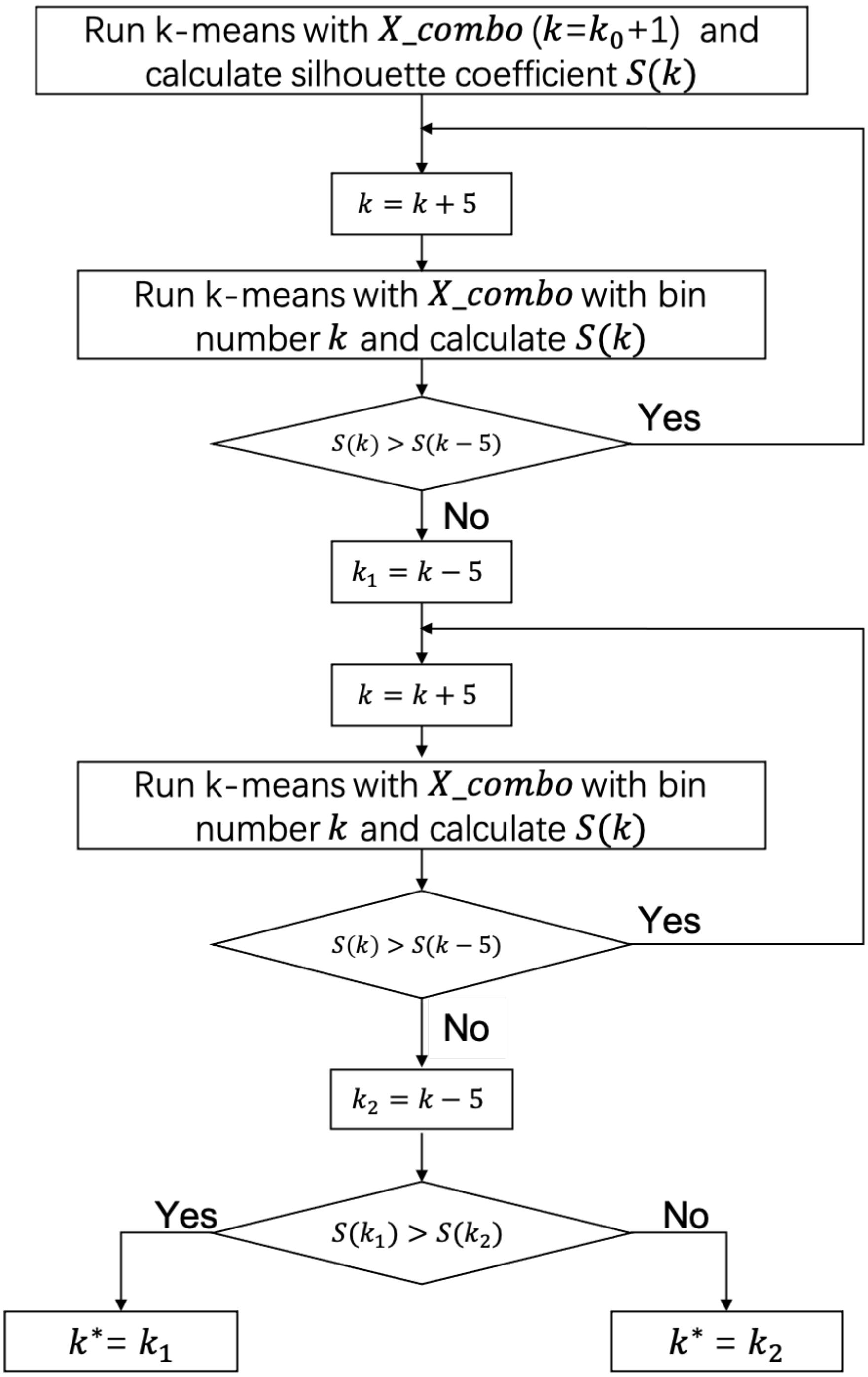

Fig. S1: The workflow of estimating the number of bins from k_0.

D Supplementary figure

E Supplementary tables

Table S1: The total number of predicted bins per binner.

Methods	CAMI Airways	CAMI Gastrointestinal tract	CAMI Mouse gut	STEC (MEGAHIT assembly)	STEC (metaSPAdes assembly)
CONCOCT	202	155	353	331	312
MaxBin	437	205	874	303	283
MetaBAT	210	158	597	218	218
VAMB	344	217	641	256	269
BMC3C	1560	342	565	921	800
DAS Tool	137	156	556	140	105
MetaWRAP	144	139	523	155	143
MetaBinner	285	202	641	194	167

Table S2: Performance comparison of the binners on the real dataset evaluated by CheckM.

Dataset	Methods	Metrics					
		#bins (>50% comp <10% cont)	#bins (>70% comp <10% cont)	#bins (>90% comp <10% cont)	#bins (>50% comp <5% cont)	#bins (>70% comp <5% cont)	#bins (>90% comp <5% cont)
STEC (metaSPAdes assembly)	CONCOCT	101	72	22	92	65	21
	MaxBin	102	72	*28*	86	61	*26*
	MetaBAT	91	53	18	87	50	18
	VAMB	*129*	*81*	25	*127*	*79*	25
	BMC3C	108	65	21	107	65	21
	DAS Tool	78	57	24	73	52	24
	MetaWRAP	143	96	31	135	92	31
	MetaBinner	**147**	**101**	**33**	**141**	**96**	**32**

The best results among all the methods are in bold, while the best results among the individual binners are italicized. The input binning results of MetaWRAP are generated by CONCOCT, MaxBin and MetaBAT. "#bins (>50% comp <10% cont)" denotes that the number of recovered bins that have >50% completeness and <10% contamination.

Table S3: Performance comparison of the binners on the real dataset evaluated by AMBER (based on the contigs labeled on the species-level).

Dataset	Methods	Metrics					
		#bins (>50% comp <10% cont)	#bins (>70% comp <10% cont)	#bins (>90% comp <10% cont)	#bins (>50% comp <5% cont)	#bins (>70% comp <5% cont)	#bins (>90% comp <5% cont)
STEC (MEGAHIT assembly)	CONCOCT	51	45	18	46	41	17
	MaxBin	47	40	24	37	31	19
	MetaBAT	47	24	6	44	22	5
	VAMB	57	**47**	20	50	**42**	19
	BMC3C	**61**	43	21	**54**	39	18
	DAS Tool	37	32	14	31	28	12
	MetaWRAP	53	39	11	49	36	10
	MetaBinner	50	44	**25**	46	41	**24**

The best results among all the methods are in bold. The input binning results of MetaWRAP and DAS Tool are generated by CONCOCT, MaxBin and MetaBAT. "#bins (>50% comp <10% cont)" denotes that the number of recovered bins that have >50% completeness and <10% contamination.

Table S4: Performance comparison of the binners on the CAMI Airways dataset evaluated by AMBER.

Dataset	Methods	Metrics					
		#bins (>50% comp <10% cont)	#bins (>70% comp <10% cont)	#bins (>90% comp <10% cont)	#bins (>50% comp <5% cont)	#bins (>70% comp <5% cont)	#bins (>90% comp <5% cont)
CAMI Airways	MetaBinner (post_process: mincomp_50_mincont_10)	217	196	140	186	172	125
	MetaBinner (post_process: mincomp_50_mincont_30)	215	191	142	186	169	127
	MetaBinner (post_process: mincomp_50_mincont_50)	215	191	144	186	169	129
	MetaBinner (post_process: mincomp_70_mincont_10)	217	196	140	186	172	125
	MetaBinner (post_process: mincomp_70_mincont_30)	215	191	142	186	169	127
	MetaBinner (post_process: mincomp_70_mincont_50)	215	191	144	186	169	129

"#bins (>50% comp <10% cont)" denotes the number of recovered bins that have >50% completeness and <10% contamination. "mincomp_a_mincont_b" denotes that we split bins with the contamination ($>= b$%) and completeness ($>= a$%) in Step 4.

F Protocols of the compared methods

F.1 Commands for Binning:

CONCOCT, MaxBin and MetaBAT:
metawrap binning -o path_to_outdir/INITIAL_BINNING -t 40 -a contig_file --interleaved --universal --metabat2 --maxbin2 --concoct path_to_reads/*fastq

METAWRAP:
metawrap bin_refinement -o path_to_outdir/metawrap -t 40 -A path_to_outdir/INITIAL_BINNING/metabat2_bins/ -B path_to_outdir/INITIAL_BINNING/maxbin2_bins/ -C path_to_outdir/INITIAL_BINNING/concoct_bins/ -c 50 -x 10

DAS tool:
DAS_Tool -i path_to_outdir/metawrap/concoct_bins.contigs, path_to_outdir/metawrap/maxbin2_bins.contigs, path_to_outdir/metawrap/metabat2_bins.contigs -l concoct,maxbin,metabat -c contig_file -o path_to_outdir/das_tool --threads 40 (--search_engine diamond)

*It is necessary to add the parameter "--search_engine diamond" for running DAS tool on the CAMI mouse gut dataset.

VAMB:
vamb --outdir path_to_outdir/vamb --fasta contig_file --jgi path_to_outdir/INITIAL_BINNING/work_files/metabat_depth.txt --minfasta 200000

MetaBinner:
Generate k-mer profiles:
python path_to_metabinner/scripts/gen_kmer.py contig_file 1000 4

Generate coverage profiles:
cat path_to_outdir/INITIAL_BINNING/work_files/mb2_master_depth.txt | cut -f -1,4- > coverage_profile.tsv

bash run_metabinner.sh -a contig_file -o output_dir -d coverage_profile.tsv -k kmer_profile -p metabinner_path

BMC3C:
We ran BMC3C according to the commands given in the link provided by its authors: http://mlda.swu.edu.cn/upload/code/BMC3C_README.txt

F.2 Commands for the assembly of the STEC dataset:

cat path_to_fq_files/ERR*_1.fastq > path_to_fq_files/ALL_READS_1.fastq
cat path_to_fq_files/ERR*_2.fastq > path_to_fq_files/ALL_READS_2.fastq

MetaSPAdes:
metawrap assembly --metaspades -1 path_to_fq_files/ALL_READS_1.fastq -2 path_to_fq_files/ALL_READS_2.fastq -m 900 -t 45 -o path_to_outdir/ASSEMBLY_metaspades

MEGAHIT:
metawrap assembly -1 path_to_fq_files/ALL_READS_1.fastq -2 path_to_fq_files/ALL_READS_2.fastq -m 900 -t 45 -o path_to_outdir/ASSEMBLY_megahit
